# The Paradox of Senescence in Glioblastoma: SASP as an Emerging Cancer Hallmark

**DOI:** 10.3390/cancers18040550

**Published:** 2026-02-08

**Authors:** Wataru Tarumi, Kiyohito Murai, Yasukazu Nakahata, Kenta Masui

**Affiliations:** 1 Department of Integrative Neuroscience, Graduate School of Biomedical Sciences, Nagasaki University, Nagasaki 852-8523, Japanyasu-nakahata@nagasaki-u.ac.jp (Y.N.); 2 Department of Pathology, Tokyo Women’s Medical University, Shinjuku, Tokyo 162-8666, Japan

**Keywords:** glioblastoma, SASP, therapy-induced senescence, glioma stem-like cells, inflammatory–metabolic coupling, senolytics, senomorphics

## Abstract

Cellular senescence has been traditionally viewed as a tumor-suppressive program that halts its proliferation in response to oncogenic stress or DNA damage. However, recent studies have highlighted a paradoxical role for senescence in glioblastoma (GBM), IDH-wildtype, the most aggressive primary brain tumor in adults. Accumulating evidence indicates that senescence represents a “frequent and durable” cell fate in GBM, particularly following standard therapies such as temozolomide and radiotherapy. Senescent cells frequently persist after temozolomide or radiotherapy and acquire a senescence-associated secretory phenotype (SASP) composed of inflammatory cytokines, growth factors and matrix-remodeling enzymes. These factors not only promote tumor cell survival with stemness-induction but also reshape the pro-tumorigenic microenvironment with metabolic rewiring and immune evasion. Notably, senescence also arises in non-malignant cells—including astrocytes, endothelial cells, microglia, and infiltrating immune cells—creating a multicellular senescent niche that fuels recurrence. Here, we describe a recent advance in our understanding of senescence and SASP in the pathobiology of GBM. We further focus on a state-of-the-art, challenging exploration of the idea that single-cell and spatial profiling, capable of identifying senescence- and SASP-associated morphologic and heterogeneous states, will further refine patient selection and therapeutic timing. By reframing senescence as a modifiable determinant of GBM evolution, this review underscores its emerging significance as both a cancer hallmark and a therapeutic vulnerability.

## Simple Summary

Cellular senescence is classically considered as a defense against cancer, but in glioblastoma (GBM), it can paradoxically promote tumor progression. After standard therapies such as temozolomide or radiotherapy, senescent tumor and non-tumor cells often persist and release inflammatory factors known as the senescence-associated secretory phenotype (SASP). These signals support tumor cell survival, stemness, immune evasion, and recurrence by shaping a pro-tumor microenvironment. Recent advances in single-cell sequencing and spatial transcriptomics approaches are improving the detection of senescent states, highlighting senescence as a therapeutic vulnerability in GBM.

## 1. Introduction

Cellular senescence has long been recognized as a potent tumor-suppressive mechanism, enforcing durable proliferative arrest in response to oncogenic signaling, DNA damage, or cytotoxic stress [1,2]. In normal tissues, this program functions as a critical barrier against malignant transformation by eliminating or restraining genomically unstable cells. Yet, in cancer, senescence is increasingly understood as a double-edged sword—particularly in glioblastoma (GBM), isocitrate dehydrogenase (IDH)-wildtype, the most common and lethal primary malignant brain tumor in adults. GBM is characterized by profound intratumoral heterogeneity, diffuse infiltration, metabolic and epigenetic plasticity, and near-universal recurrence despite maximal therapy [3,4,5]. These biological features create a unique microenvironment in which senescent cells often persist rather than being cleared.

Paradoxically, senescent tumor and stromal cells can acquire a robust senescence-associated secretory phenotype (SASP), consisting of pro-inflammatory cytokines, chemokines, growth factors and extracellular matrix-modifying enzymes [6,7]. In GBM, SASP factors have been shown to amplify chronic inflammation, promote mesenchymal transition and cellular plasticity, enhance immunosuppression, and foster a milieu that supports invasion and treatment resistance [8,9]. This duality underscores a central paradox: while senescence limits cellular proliferation, SASP can actively reshape the GBM ecosystem in ways that potentiate tumor aggressiveness. Of interest, senescence/SASP could play a role in the biology of genotypically different brain tumors, including IDH-mutant gliomas [10], and exploration of this paradox in cancer cell senescence would have a broader impact on the clarification of brain tumor pathobiology.

Clinical therapies used against GBM—including radiotherapy, temozolomide, and various targeted agents—are themselves potent inducers of therapy-associated senescence [11]. Accumulating evidence suggests that such therapy-induced senescence may inadvertently promote tumor progression through SASP-mediated signaling, contributing to recurrence and resistance. As a result, deciphering the molecular determinants of SASP in GBM has become a priority, opening avenues for senolytic strategies that selectively eliminate senescent cells or senomorphic approaches that suppress SASP without reversing senescence [12,13].

In this review, we synthesize current insights into the multifaceted roles of senescence in GBM, emphasizing the tumor-promoting activities of SASP and their implications for therapeutic resistance and tumor evolution. By reframing senescence as a dynamic and context-dependent process rather than a terminal cellular fate, we aim to highlight emerging vulnerabilities that may inform the next generation of GBM therapies.

## 2. Basic Concepts and Dual Roles of Cellular Senescence in Tumorigenesis

Cellular senescence is a stable and essentially irreversible state of growth arrest that cells enter in response to a variety of intrinsic and extrinsic stresses [14]. The concept was first articulated by Hayflick and Moorhead in 1961, who demonstrated that human fibroblasts possess a finite replicative lifespan—now known as replicative senescence [15]. Subsequent work has revealed that senescence can also be triggered independently of telomere erosion by DNA damage, oxidative stress, chromatin perturbation, and oncogene activation [1,16]. These stressors activate a sustained DNA damage response (DDR), which enforces growth arrest primarily through the p53–p21 and p16INK4a–Rb tumor suppressor pathways [17,18].

Although senescent cells are no longer capable of proliferation, they remain metabolically active and undergo profound transcriptional and secretory reprogramming. A defining feature of this state is the senescence-associated secretory phenotype (SASP), a complex mixture of pro-inflammatory cytokines (e.g., IL-6, IL-8), growth factors (e.g., VEGF, HGF), chemokines and matrix-remodeling enzymes such as matrix metalloproteinases (MMPs) [6]. Associated with actively reprogrammed metabolomic features in cancer [19], the broader repertoire of SASPs including extracellular vesicles, non-coding RNA, bioactive lipids, and other mediators, could be referred to as the “SASPome” [20], collectively framing the tumor microenvironment to propagate senescence signals, modulate inflammation, and remodel extracellular architecture. Through SASP, senescent cells can thus influence the behavior of neighboring cells and remodel the local tissue environment, extending their impact far beyond cell-autonomous growth arrest.

**Figure 1:**
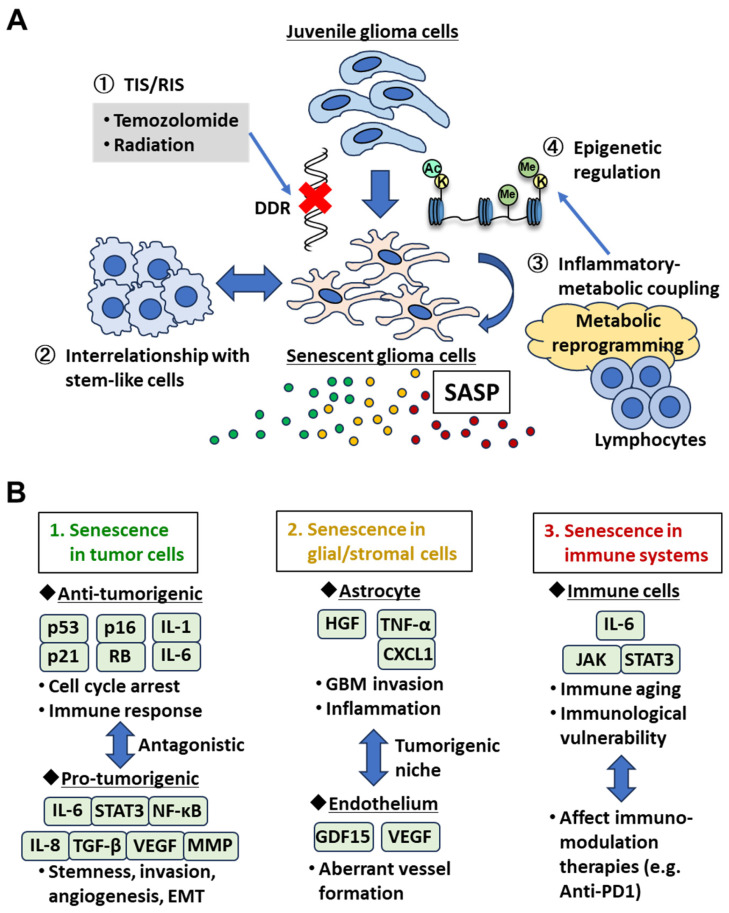
Induction, maintenance and consequences of senescence/SASP in GBM. (**A**) Induction and maintenance of senescence/SASP in GBM. Actively proliferating glioma cells could be induced and maintained in the senescent state with SASP by ① the effect of chemotherapeutics and radiation (TIS), ② the interaction of glioma stem-like cells, ③ inflammatory–metabolic coupling and ④ epigenetic regulation. (**B**) Consequences of senescence/SASP in GBM. Senescence in GBM cells shows anti-tumorigenic effects including cell cycle arrest and immune reaction, whereas it exerts pro-tumorigenic effects such as stemness, invasion, angiogenesis, and epithelial–mesenchymal transition (EMT). Senescence in glial/stromal cells creates a tumorigenic niche where senescent astrocytes promote tumor cell invasion and inflammation, and senescent endothelial cells contribute to abnormal vessel formation. Further, senescence in immune systems relates to immunological vulnerabilities and affects the effect of immune checkpoint inhibitors.

Senescence and SASP exhibit strikingly dualistic roles in tumorigenesis and tumorigenic niche and immune microenvironments (Figure 1). In early stages, senescence acts as a robust tumor-suppressive barrier. For example, premalignant lesions often accumulate p16INK4a-positive senescent cells that prevent further clonal expansion and block malignant transformation [21]. Oncogene-induced senescence (OIS), triggered by aberrant activation of oncogenes such as RAS or BRAF, represents a key mechanism through which tissues restrain early neoplastic growth [1]. However, when senescent cells persist long-term—often due to impaired immune clearance—the SASP can become maladaptive. Chronic secretion of SASP factors can establish a pro-tumorigenic inflammatory milieu that enhances tumor growth, invasion, angiogenesis, and tissue remodeling [6,22]. In this context, SASP shifts from being a guardian against malignant transformation to a promoter of tumor progression. The important question is at what point a senescent cell switches from tumor suppressor to tumorigenic, but there is no clear evidence of a uniform switch point on it. Future studies are thus needed to define the threshold and dynamic switch between tumor-suppressive senescence and tumor-promoting SASP states.

Thus, cellular senescence represents a time- and context-dependent biological process with biphasic effects: acutely tumor-suppressive yet chronically tumor-promoting [23]. This temporal transition highlights the “clock-like” nature of senescence biology, wherein the duration, persistence, and clearance of senescent cells critically shape their overall impact on tumorigenesis. In the following sections, we discuss the role of aging in GBM biology using GBM models, but no single marker is sufficient to define bona fide senescence, and careful interpretation with multiple markers is required (Table 1).

## 3. Mechanistic Insight into Cellular Senescence in GBM

### 3.1. Therapy-Induced Senescence as a Major Cell Fate in GBM

**Figure 2:**
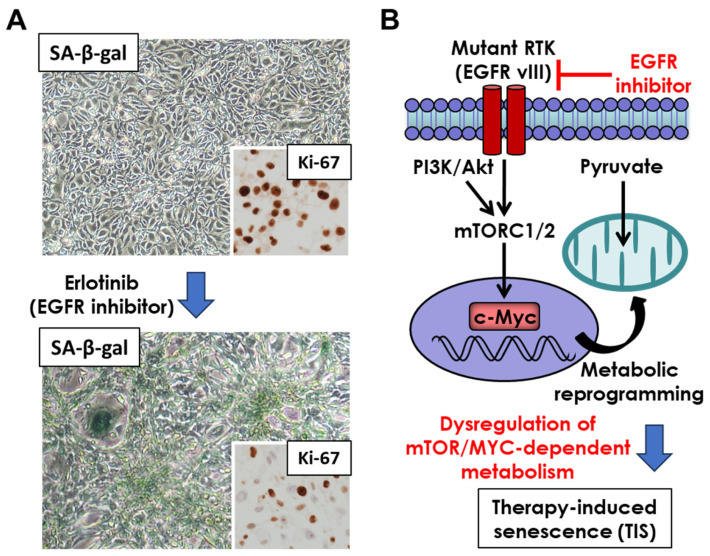
Therapy-induced senescence of GBM cells by EGFR inhibitors. (**A**) U87 GBM cells expressing mutant/amplified forms of EGFR (EGFRvIII) are treated by the EGFR inhibitor (erlotinib: first generation EGFR-TKI). Erlotinib treatment induces senescence of GBM cells with morphological changes in plump cytoplasm, reduction in cell proliferation (Ki-67) and expression of the senescence marker SA-β-gal. (**B**) EGFR inhibitors could induce senescence in GBM cells, potentially by interfering with oncogene (e.g., mTOR, c-Myc)-dependent metabolic reprogramming in tumor cells. These demonstrate an important intersection of oncogene signaling, metabolic reprogramming and senescence, induced by molecular targeting therapies.

GBM is among the most treatment-refractory human cancers, and the current standard regimen—surgery followed by radiotherapy and temozolomide (TMZ)—provides only modest survival benefit [3]. A growing body of evidence indicates that these therapies frequently induce therapy-induced senescence (TIS) in tumor and stromal compartments potentially via DDR pathways (Figure 1A), forming a long-lived population of non-proliferative but metabolically active cells rather than simply eliminating tumor cells [24,25]. Irradiated GBM cells frequently undergo persistent DNA damage and display supportive senescence hallmarks such as metabolic and lysosomal remodeling, which is commonly detected as senescence-associated β-galactosidase (SA-β-gal) activity. Importantly, despite extensive DNA damage, senescent cells acquire resistance to apoptosis through the upregulation of anti-apoptotic BCL-2 family proteins, particularly BCL-XL. This BCL-XL-dependent survival enables senescent cells to persist during and after therapy, contributing to altered cell turnover and sustained SASP signaling within the tumor microenvironment [25]. TMZ induces an analogous phenotype. Aasland et al. (2019) showed that TMZ activates ATR–Chk1 signaling, causing replication stress, downregulation of DNA repair pathways and a stable growth-arrested state [26]. Importantly, senescent GBM cells remain transcriptionally active, secreting factors such as IL-6 that engage the JAK2–STAT3 pathway in neighboring non-senescent cells [27]. Recent work by Wang et al. (2025) indicates that TMZ-induced senescence is coupled to a hypermetabolic state, driven by HIF-1α/HIF-2α [28]. Dual knockout of HIF1α/HIF2α suppressed SA-β-gal activity, reduced SASP gene expression (IL-1A, IL-1B, IL-6, IL-8, CCL2, ATM), and prevented the emergence of post-senescent progeny. Notably, these senescent cells could eventually regain proliferative capacity and produced progeny with enhanced stemness, invasiveness and TMZ resistance, associated with reprogramming of the metabolism and the pro-tumor immune microenvironment in GBM with TIS phenotypes [28,29]. Of interest, molecular-targeting therapies, including EGFR inhibitors, could also induce senescent phenotypes including morphological changes, SA-β-gal expression and low proliferative activities in GBM [30,31,32], supported by our dataset (Figure 2A), which should be clinically important since EGFR amplification is one of the most common genetic aberrations in GBM, being found in approximately 40% of patients [33]. Thus, TIS in GBM can represent an intermediate and plastic cell fate that is capable of evolving into more malignant states. Collectively, these findings suggest that standard therapy and molecular therapeutics inadvertently generate a persistent senescent compartment that actively shapes the tumor environment.

### 3.2. Growth-Arrested States Within GBM Stem-like Compartments

GBM harbors heterogeneous stem-like populations (e.g., CD133^+^, SOX2^+^) that exhibit variable sensitivity to genotoxic stress [34]. Early reports proposed that CD133^+^ glioma stem-like cells (GSCs) are intrinsically therapy-resistant [35,36], but subsequent studies demonstrated the opposite. For example, Chang et al. (2009) showed that patient-derived CD133^+^ tumor stem cells had impaired DNA double-strand break repair and were more radiosensitive than standard GBM cell lines [37]. Functional heterogeneity is further underscored by the finding that SOX2 is essential for tumor-initiating capacity [38]. This heterogeneous, therapeutically resistant or sensitive nature of GSCs is reminiscent of tumor cells with dual characteristics of senescence or SASP [39,40], although the clarification of the link between the two states needs further examination of both aging markers and stem cell markers in these population. Indeed, although classical senescence within purified GSC fractions has not been fully characterized, therapy produces a spectrum of growth-arrested states—including senescence, dormancy and quiescence—in which stem-like properties persist. Long-term TMZ exposure generates dormant, slow-cycling GBM cells that co-express dormancy markers (IGFBP5, EphA5, H2BK) along with SOX2, OCT4 and KLF4 [41]. These observations suggest that therapy does not eliminate stemness, but could instead enrich or preserve it within non-proliferative populations, associated with senescence, dormancy and quiescence (Figure 1A). Conversely, the concept of senescence-associated reprogramming is also supported by Wang et al. (2025), who showed that TMZ-induced senescent cells could facilitate HIF-dependent transitions toward stem-like states [28]. In line with this, single-cell transcriptomic studies identified slow-cycling, therapy-resistant GBM populations with reduced proliferation markers and enhanced lineage plasticity [42,43]. Although not explicitly labeled as senescent, these clusters share features—stress adaptation, metabolic rewiring and transcriptional plasticity—consistent with senescence-like states. Far more complicatedly, senescence from GSC differentiation promotes tumor growth in part via the paracrine effects of the secreted proteins [44], suggesting a tight link between senescent phenotype and cancer stem cell state. Together, these findings suggest that therapy generates a continuum of non-proliferative but viable cell states, within which stem-like programs and growth-arrested states can bidirectionally interact, providing a pro-tumorigenic reservoir for GBM cells with senescence, dormancy and quiescence. One of the caveats here is regarding terminology. Although senescence, quiescence, and dormancy are all characterized by growth arrest, they represent biologically distinct states. Senescence is defined by a durable cell cycle exit accompanied by persistent DNA damage signaling and sustained SASP production, whereas quiescence is a reversible, non-damaging G0 state lacking SASP [2,45]. Tumor dormancy encompasses a broader spectrum of non-proliferative states and may include quiescent or senescent cells depending on microenvironmental cues [46]. Given that many of the central conclusions of this review rely on SASP-mediated effects, we restrict the use of the term senescence to studies demonstrating sustained SASP or multiple orthogonal senescence markers.

### 3.3. Inflammatory–Metabolic Coupling as a Basis for Senescence and SASP Maintenance in GBM

Senescent cells could be induced by profound inflammatory and metabolic remodeling [47] (Figure 1A). A central feature is mitochondrial dysfunction and persistent ROS accumulation, which reinforces senescence through the p21–ROS–DNA damage loop described by Passos et al. [48]. Persistent DNA damage signaling activates NF-κB, driving transcription of SASP genes such as IL-6 and IL-8 [49,50]. These pathways form a reinforcing feedback network that stabilizes both senescence and SASP expression. GBM TIS shares these features. Radiation and TMZ induce DNA damage, ROS and NF-κB activation, suggesting comparable feedback architecture [51]. The full continuity of the p21–ROS–DDR–NF-κB–SASP axis in GBM remains to be directly demonstrated, but the available evidence supports a working model in which oxidative stress and inflammatory signaling sustain SASP expression. Senescence in GBM also involves distinct metabolic features, and metabolic shift is one of the cardinal hallmarks of cancer. We demonstrated that EGFR signaling drives cancer metabolism in a Myc-dependent manner [52], the inhibition of which could induce the senescent phenotypes in GBM cells, possibly through the dysregulation of mTOR- and Myc-dependent metabolism [53,54] (Figure 2B). Furthermore, IL-6 from TIS cells promotes bystander senescence via JAK–STAT3–NF-κB signaling [27,55], and STAT3 additionally upregulates glycolytic enzymes, linking SASP signaling to a Warburg-like metabolism [56,57]. Sustained senescence is energetically costly. Senescent cells exhibit low amounts of NAD^+^ due to the decrease in nicotimamide phosphoribosyltransferase (NAMPT) mRNA and protein [58,59] and increase NAD^+^ consumption due to persistent DNA repair and SASP gene transcription. Nacarelli et al. (2019) demonstrated that some cancer-associated senescent cells rely on the NAMPT-mediated NAD^+^ salvage pathway, as well as proliferating cells [60]. In GBM, the NAMPT–NAD^+^ axis broadly supports tumor survival and transcriptional programs [61], suggesting that TIS cells may be especially vulnerable to NAD^+^ depletion. Additionally, NAD^+^ depletion with NAMPT inhibitors could be a promising strategy against IDH-mutant gliomas [62], and the metabolism-SASP correlation should have a broader impact on the brain tumor biology. Senescence-associated metabolic reprogramming extends to immune cells. De Leo et al. showed that monocyte-derived macrophages in GBM adopt a glycolytic phenotype that produces intracellular lactate, which induces p300-mediated H3K18 lactylation and upregulates IL-10, promoting immunosuppression [63]. In addition, slow-cycling GBM cells preferentially rely on lipid metabolism and reprogram T cell metabolism from glycolysis to fatty acid oxidation, leading to T cell senescence and diminished cytolytic activity [64]. This highlights that tumor-derived and immune-cell-derived metabolites function to reinforce a senescence-associated, immunosuppressive niche. Overall, these findings support a model in which inflammation and metabolism are tightly coupled in senescent GBM cells. This integrated network stabilizes senescence, sustains SASP, and modifies the microenvironment in ways that influence therapeutic outcomes.

### 3.4. Epigenome-Dependent Regulation of Senescence and SASP in GBM

Epigenetic alterations constitute a central layer of regulation that locks GBM cells into a senescent state and modulates SASP output [65] (Figure 1A). Senescent glioma cells undergo characteristic chromatin remodeling, including the loss of H3K27me3, focal H3K9me3 accumulation, and the formation of senescence-associated heterochromatin foci (SAHF), which is a unique heterochromatin structure used as a marker of senescence and associated with a number of effectors in the senescence program [66]. A shift in histone marks and chromatin compaction stabilize cell cycle arrest while permitting selective activation of inflammatory loci [67,68]. These changes are mediated by histone modifiers such as KDM6A/B and EZH2, as well as DNA methylation-dependent mechanisms that shape enhancer accessibility and NF-κB-driven SASP transcription. Importantly, GBM exhibits profound DNA methylation drift with aging, and emerging evidence suggests that senescent tumor cells partially converge on “epigenetic clock” signatures originally described in normal aging tissues [69,70]. Such clock-like methylation patterns, including age-associated hypomethylation and promoter hypermethylation at Polycomb-regulated genes, may sensitize GBM cells to senescence-inducing stresses and influence the magnitude of SASP. In addition to the epigenetic regulation of senescent phenotypes in cancer cells, aging of epigenomes itself, “epigenetic senescence” is also at the intersection of aging and tumor formation for GBM [71]. Although the role of epigenetic clocks in tumor senescence is only beginning to be explored, integration of methylation-based biological age with transcriptional senescence markers may provide a powerful framework to understand intertumoral variability in GBM senescence programs [72,73].

## 4. Functional Consequences of Senescence and SASP in GBM

Cellular senescence in GBM—whether occurring in tumor cells, glial/stromal populations, or immune components—acts not merely as terminal growth arrest but as a dynamic state that reshapes the tumor ecosystem through SASP-driven signaling (Figure 1B). We outline below how senescence influences malignant progression through distinct but interconnected cellular compartments.

### 4.1. Senescence in Tumor Cells Provides Dual Pathways for GBM Formation

Cellular senescence in GBM functionally contributes to tumor suppression [74]. Intrinsically, senescence is triggered by DNA damage from radiotherapy or TMZ (i.e., TIS, RIS), oncogenic signaling (i.e., OIS by aberrant RTK/RAS/PI3K activation, etc.), and telomere dysfunction, which converge on the p53–p21 and p16INK4A–RB pathways to impose a stable cell cycle arrest, thus suppressing tumor cell proliferation [8]. In GBM, even partially functional DDR signaling can induce a senescent-like state characterized by persistent γ-H2AX foci, heterochromatin reorganization (SAHF-like domains), and metabolic rewiring such as decreased oxidative phosphorylation and increased lysosomal activity [75]. These data show that cellular senescence has an anti-tumorigenic effect for GBM formation.

Conversely, senescence and SASP could also mechanistically contribute to tumor promotion in GBM [8]. Senescent GBM cells and stromal elements (astrocytes, endothelial cells, microglia) release IL-6/STAT3-activating cytokines, IL-8/CXCR1/2 chemokines, TGF-β, VEGF, and MMPs, collectively implicated in tumor cell stemness, invasion, angiogenesis, and EMT-like transitions [8,27,40]. Further, TIS cells can re-enter the cell cycle through SASP-dependent “senescence escape” pathways, involving downregulation of p21, chromatin remodeling, and acquisition of a more stem-like transcriptional program [76], enabling highly tumorigenic states upon escape [77]. Moreover, Dou and Berger (2018) similarly proposed that senescence and stemness share regulatory chromatin architecture, positioning senescence as a fertile ground for plasticity [78]. Notably, SASP-mediated activation of STAT3, NF-κB, and C/EBPβ is associated with the tumorigenic immunosuppressive microenvironment [79] and remodeling of the extracellular matrix to enhance TMZ and radiation resistance [25], which will be discussed later. Overall, the available evidence suggests that senescence can play dual roles in tumor formation of GBM both directly and through microenvironmental cues.

### 4.2. Senescence in Glial and Stromal Cells Constitutes Specific Niche for GBM Cells

TIS extends beyond malignant cells and generates a multicellular senescent niche with profound consequences for tumor behavior [80]. Irradiated astrocytes acquire canonical senescence features and secrete HGF, promoting MET-dependent invasion in GBM cells [81]. In vivo, Ji et al. (2024) identified extensive senescent astrocyte accumulation especially at the tumor margin/periphery, and showed that their SASP—particularly TNF-α—induces CXCL1 production in GBM cells via Myc–Max signaling, driving recruitment of CXCR2^+^ myeloid cells and fostering tumor regrowth [80]. In the hypoxic region (usually at the necrotic/perinecrotic core), hypoxia-induced reactivity of tumor-associated astrocytes affects glioma cell properties partially through SASP-like phenotypes [82]. Endothelial senescence represents another critical component of this niche. Mechanistic insights from vascular biology indicate that oxidative stress and GDF15 promote p16-dependent endothelial senescence [83]. Subsequent work in GBM demonstrated that irradiated brain endothelial cells secrete GDF15, triggering VEGFA upregulation in tumor cells through MAPK1/SP1 signaling and promoting abnormal angiogenic sprouting [84]. Such structurally aberrant vessels likely disrupt perivascular integrity and may facilitate GBM infiltration. Far more interestingly, in GBM recurring after radiotherapy, where the brain endothelium suffers from radiation-induced cell senescence, tumor-derived endothelium plays a role in neo-vascularization [85]. Single-nucleus RNA sequencing by Dehghan et al. (2024) further showed that irradiation induces senescence-associated programs across multiple cell types—including astrocytes, oligodendrocyte-lineage cells, microglia, and endothelial subsets—indicating that therapy shapes the GBM microenvironment through the coordinated induction of senescence [25]. Of interest, senescence/SASP networks play a role in the communication among non-neoplastic cells [86], which may create a pro-tumorigenic niche, including the interface of astrocytes and blood vessels as blood–brain barriers (BBB). Together, these findings indicate that senescence in glial and stromal cells acts as an active organizer of microenvironmental remodeling, generating a SASP-rich niche that could facilitate cellular proliferation, invasion, and angiogenesis. Since recent reports clearly demonstrated that aggressiveness in GBM cells derives from their interaction with residing non-neoplastic cells at the invasive front of the tumor–brain interface [72,87], the interaction among GBM cells and non-neoplastic cells, including neuron, glia and stromal cells, should be actively explored from a senescent standpoint.

### 4.3. Senescence in the Immune System Contributes to GBM-Related Immune Microenvironment

Senescent tumor and stromal cells significantly reprogram the immune landscape through SASP-mediated signaling [29,40]. Senescent tumor cells can adopt a “senescence surveillance” phenotype in which SASP components (e.g., CCL2, CXCL10, IL-6/IL-1-dependent cytokine networks) promote the recruitment of NK cells, CD8+ T cells, and inflammatory macrophages, and TIS in GBM can upregulate NKG2D ligands and antigen presentation machinery, thereby sensitizing tumor cells to cytotoxic lymphocytes and contributing to a temporary window of vulnerability following genotoxic stress [29,88]. Conversely, senescent astrocytes influence immune composition by secreting CXCL1, which recruits myeloid-derived cells and establishes an immunosuppressive environment that promotes recurrence. Ji et al. further demonstrated that removing senescent astrocytes diminished immune infiltration and delayed tumor regrowth, highlighting their functional importance [80]. Notably, immune cells themselves should age, and senescence in immune cells refers to a permanent cessation of the cell cycle accompanied by the expression of distinct hallmarks of senescence [89]. Eventually, cellular senescence is implicated in distinct immunological vulnerabilities in cancer [90]. On the other side of immunological aspects, senescence in the immune system could affect the efficiency in immunomodulation therapies, including anti-PD1/PD-L1 [91]. Using single-cell transcriptomic analysis, another study could reverse the senescence-associated, exacerbated immunosuppressive profile of myeloid cells in the tumor microenvironment, and the elimination of these myeloid cells restores CD8 T cell proliferation and abrogates immunotherapy resistance [92]. This further supports the idea that the use of senolytic drugs before immune checkpoint inhibitors (ICIs) may constitute a pharmacological approach to improve the effectiveness of cancer immunotherapies.

Collectively, these observations converge on a unifying principle: SASP could play a role as a central hub connecting inflammatory, vascular, and immune pathways. Through regulators such as NF-κB and STAT3, senescent cells orchestrate chronic inflammation, recruit suppressive immune subsets, and create conditions favorable to tumor persistence. Rather than passive byproducts of therapy, senescent cells represent a potential contributor to a pro-tumorigenic immune milieu.

## 5. Therapeutic Targeting of the Senescence Network in GBM

Conventional cytotoxic therapies such as radiation and TMZ aim to eradicate malignant cells but frequently induce TIS [93]. Senescent cells that persist after treatment remain metabolically active and release SASP factors that alter immune, stromal, and vascular components of the tumor microenvironment—ultimately promoting recurrence. As a result, senescence and SASP have emerged as therapeutically actionable processes rather than passive byproducts of DNA-damaging therapy (Table 2).

### 5.1. Senolytic Strategies: Eliminating Senescent Cells

Senolytics are designed to selectively kill senescent cells by targeting senescence-associated anti-apoptotic pathways (SCAPs) [102]. Several recent studies highlight their potential relevance in GBM. Rahman et al. (2020) demonstrated that irradiated, senescent GBM cells upregulate BCL-XL and rely on this protein for survival [94]. Notably, knockdown of BCL-XL decreased the survival of radiated GBM cells, whereas knockdown of BCL-2 or BCL-W yielded no senolytic effect. Pharmacologic inhibition with navitoclax (a potent and orally bioavailable BCL-XL/BCL-2 inhibitor) eliminated senescence, but not the proliferation of GBM cells, revealing a vulnerability that emerges specifically in the senescent state. Navitoclax has thus demonstrated senolytic activity in multiple peripheral tumor models; however, its ability to achieve therapeutically relevant concentrations within the central nervous system (CNS) remains uncertain. While BBB disruption in the tumor core may permit partial drug penetration, infiltrative glioma cells often reside behind an intact BBB, posing a major translational challenge [103]. Similarly, Tomimatsu et al. (2025) identified the NF-κB–cIAP2 axis as another SCAP module; inhibiting cIAP2 with birinapant efficiently triggered apoptosis in senescent GBM cells [95]. The therapeutic implications extend beyond tumor cells. Fletcher-Sananikone et al. (2021) showed that eliminating senescent astrocytes after irradiation reduced GBM invasion and slowed tumor growth, illustrating how senolytics can remodel the broader senescent microenvironment [81]. Beyond BCL-XL and cIAP2, additional classes of senolytics are under investigation—such as FOXO4-DRI peptides [96] and fisetin-/quercetin-based compounds [97]—which may provide alternative or synergistic routes for senescent cell clearance in the CNS, though direct evidence supporting its efficacy in intracranial tumor models is limited since their brain penetrance remains to be improved as a peptide-based agent.

An additional concern for senolytic therapy in GBM is the potential on-target toxicity toward normal senescent-like populations in the brain by aging and radiation, such as astrocytes and neural progenitor cells [104]. Given the role of these cells in neurogenesis and tissue homeostasis, indiscriminate elimination of senescent cells may carry risks of cognitive or regenerative impairment.

### 5.2. Senomorphic Strategies: Modulating SASP Without Removing Senescent Cells

Because senescence-associated growth arrest also functions as a tumor-suppressive barrier, complete removal of senescent cells may carry risks. Senomorphics aim instead to suppress SASP production while preserving cell cycle arrest. Xu et al. (2015) showed that JAK1/2 inhibition markedly reduces IL-6, IL-8, and MCP-1 expression in senescent human preadipocytes, identifying JAK–STAT signaling as a principal SASP-maintaining pathway [98]. These findings have clear parallels in GBM, where IL-6–STAT3 signaling drives bystander senescence and maintains pro-tumorigenic inflammation [27,95]. Additional pathways under investigation as SASP regulators include NF-κB signaling, a master transcriptional regulator of inflammatory SASP components [49]; and mTOR signaling to promote SASP translation, in which rapamycin analogs attenuate SASP without reversing arrest [99]. Interestingly, immunosuppressive drugs such as Frentizole derivatives could be reported with mTOR-inhibiting and senomorphic properties, which disrupts disease conditions even in non-neoplastic CNS disorders including toxic amyloid β [105]. p38-MAPK is a stress–response pathway required for SASP induction [100], and targeting these pathways in GBM may blunt the inflammatory amplification loops (e.g., IL-6, TNF-α, CXCL1) driven by senescent tumor and stromal cells while retaining the cytostatic benefits of senescence. Since JAK-STAT, NF-κB and mTOR are the main aberration in GBM [33], such senomorphic approaches could be a promising therapeutic strategies against GBM from both perspectives of molecular-targeting and senescence-targeting therapeutics.

### 5.3. A Two-Phase or “Timed” Therapeutic Framework

The current evidence supports a temporally structured therapeutic approach. (1) During active chemoradiation: use senomorphics (JAK–STAT inhibitors, NF-κB inhibitors, or mTOR modulators) to suppress SASP-mediated inflammation, reduce bystander senescence, and prevent reconstruction of a pro-recurrence microenvironment; and (2) after completion of therapy: apply senolytics (BCL-XL, cIAP2, or other SCAP-targeting agents) to clear residual senescent tumor and stromal cells that contribute to relapse. There are many layers of complexity underlying the successful clinical drug implementation of what appears to be a very rational and simple biological concept. Toxicity, dose scheduling and molecular heterogeneity are issues that surround drug efficacy and resistance, and the future of the approach requires a conjoint consideration of what has been learned from clinical failures as well as successes, driven by mechanistic knowledge of therapeutic sensitivity and resistance to guide the design of rational future clinical trials with the goal of establishing novel and more effective treatment paradigms [33]. Thus, this two-phase or “timed” therapeutic approach mirrors emerging strategies in aging biology and cancer therapeutics [33,101], providing a conceptual scaffold for integrating senescence biology into GBM management.

### 5.4. Therapeutic Challenges and Future Directions

Despite promising preclinical data, several obstacles remain before senescence-directed therapies can be incorporated into GBM care: (1) Lack of specific biomarkers—Reliable markers that distinguish senescent tumor cells from slow-cycling or quiescent cells remain limited. Composite signatures combining p16INK4a, DNA damage markers, chromatin reorganization, and SASP profiles may be required. (2) Heterogeneity of SASP—SASP composition varies across cell types, treatments, and time. Detailed SASP atlasing using single-cell and spatial omics will be essential for identifying actionable nodes. (3) Timing and delivery—Optimal sequencing of senomorphics and senolytics remains unclear, and BBB penetration represents an additional challenge for many senotherapeutics. (4) Intersection with immune therapy—Senescence influences T cell exhaustion, myeloid recruitment, and antigen presentation. How senolytics or SASP suppression interact with immune checkpoint blockade or myeloid-directed therapies remains largely unexplored. (5) Impact on neural repair—Senescent glial cells participate in wound responses and tissue remodeling [106,107]. Completely ablating these populations could impair repair mechanisms after radiation injury. Future work should also consider the temporal dynamics of SASP and GBM metabolism. Given that senescence induction and SASP output exhibit circadian variation [108], incorporating chronotherapy may improve the efficacy and reduce toxicity of senescence-targeting interventions. Taken together, repositioning senescence from a passive byproduct of therapy to a modifiable driver of tumor behavior reframes treatment opportunities. By integrating senolytic and senomorphic approaches within a temporally informed therapeutic paradigm, senescence-targeted strategies may become a central component of next-generation GBM therapy.

## 6. Conclusions

Senescence in GBM represents a far more dynamic and influential process than previously appreciated. Rather than acting solely as a cytostatic barrier, senescent tumors and glial, stromal, and immune cells cooperatively remodel the tumor ecosystem through SASP-driven inflammatory, metabolic, and vascular signaling. TIS can generate hypermetabolic and transcriptionally plastic states that foster stemness, invasion, and recurrence, while senescent astrocytes and endothelial cells create a supportive niche that amplifies immune suppression and aberrant angiogenesis. Together, these findings position senescence as a central coordinator of GBM progression [40].

Therapeutically, targeting the senescence network offers compelling opportunities. Senomorphic approaches can attenuate SASP-mediated inflammation during active therapy, whereas senolytics hold promise for post-treatment clearance of residual senescent compartments that seed recurrence. Notably, senotherapeutics could be applicable to other CNS disorders, including neurodegeneration and metabolic changes [109,110]. However, successful translation will require careful optimization of timing, biomarkers, and delivery strategies, along with a deeper understanding of senescence heterogeneity across the tumor microenvironment.

Emerging diagnostic innovations—including AI-driven digital pathology—are poised to transform this landscape. Deep-learning models trained on cellular morphology, chromatin texture, spatial architecture, and SASP-linked microenvironmental patterns may enable automated detection of senescent and SASP-producing cells in routine histology [111,112]. Integration of single-cell, spatial transcriptomic, metabolomic and epigenetic “clock” signatures into multimodal AI frameworks could provide unprecedented resolution in identifying senescence states, predicting therapy-induced senescence burden, and guiding metabolism-based, senolytic/senomorphic interventions.

Accumulating evidence indicates that direct in vivo evidence establishing causality between SASP and GBM biology remains limited. It is important to acknowledge that much of the current evidence linking SASP to GBM recurrence is derived from correlative transcriptomic analyses, cytokine profiling, and in vitro functional assays. Nevertheless, the consistency of SASP-associated signatures across independent datasets and experimental systems highlights the biological plausibility of SASP as a functional contributor to tumor persistence and warrants further mechanistic investigation in vivo.

Ultimately, repositioning senescence from an incidental consequence of therapy to a modifiable, measurable, and targetable driver of GBM biology opens new avenues for precision treatment. Advances at the interface of senescence biology, metabolism, microenvironmental analysis, and AI-powered diagnostics may collectively define the next generation of GBM therapeutic strategies.

## Figures and Tables

**Table 1:** Definition of senescence markers for GBM models.

Category	Markers	Limitations
Core	Durable growth arrest, persistent DDR, SASP	Long-term observation is required
Supportive	SA-β-gal, p16, p21, SAHF, anti-apoptotic markers	Non-specific markers
Context-dependent	Transcriptomic signatures	Correlative in nature, not causative

DDR, DNA damage response; SASP, senescence-associated secretory phenotype; SA-β-gal, senescence-associated β-galactosidase; SAHF, senescence-associated heterochromatic foci.

**Table 2:** Therapeutic strategies targeting senescence and SASP in GBM.

Strategy	Target/Pathway	Representative Agents	Relevance in GBM	Key References
**Senolytics**	BCL-XL (SCAP)	Navitoclax (ABT-263)	RIS GBM cells become dependent on BCL-XL for survival; selective elimination of TIS cells	Rahman et al. 2020 [94]
	NF-κB–cIAP2 axis	Birinapant	Senescent GBM cells rely on cIAP2-mediated apoptosis resistance	Tomimatsu et al. 2025 [95]
	Senescent astrocytes	Navitoclax	Clearance of senescent astrocytes suppresses invasion and tumor-supportive microenvironment	Fletcher-Sananikone et al. 2021 [81]
	p53–FOXO4 interaction	FOXO4-DRI peptide	Induces apoptosis selectively in senescent cells; CNS applicability under investigation	Tripathi et al. 2021 [96]
	Multi-SCAP targeting	Fisetin, Quercetin	Flavonoid-based senolytics with potential CNS activity	Spiegel M. 2025 [97]
**Senomorphics**	JAK–STAT signaling	JAK1/2 inhibitors	Suppress IL-6/STAT3-driven inflammatory SASP and bystander senescence in GBM	Xu et al. 2015 [98]
	NF-κB signaling	NF-κB inhibitors	Master regulator of pro-inflammatory SASP components	Chien et al. 2011 [49]
	mTOR signaling	Rapamycin, rapalogs	Attenuate SASP translation while preserving growth arrest	Laberge et al. 2015 [99]
	p38 MAPK	p38 inhibitors	Stress–response pathway essential for SASP induction	Freund et al. 2011 [100]
**Timed therapeutic model**	Phase I: SASP suppression	Senomorphics	During chemoradiation, limit SASP-driven inflammation and microenvironmental remodeling	Two-phase or “timed” approach Masui et al. 2014 [33] Kirkland et al. 2017 [101]
	Phase II: senescent cell clearance	Senolytics	After therapy, eliminate residual senescent tumor and stromal cells to prevent recurrence	Two-phase or “timed” approach Masui et al. 2014 [33] Kirkland et al. 2017 [101]

## Data Availability

The original contributions presented in this study are included in the article. Further inquiries can be directed to the corresponding author.
